# Metagenomic insights into the rhizosphere microbiome dysbiosis associated with tobacco bacterial wilt

**DOI:** 10.3389/fmicb.2026.1809980

**Published:** 2026-04-15

**Authors:** Ligang Xiang, Xiaoyan Wang, Mingxia Wen, Xu Wang, Yanhong Zhang, Weiqiang Tian, Minghong Liu, Wenjian Zhang

**Affiliations:** Guizhou Province Tobacco Company Zunyi Branch, Zunyi, China

**Keywords:** tobacco bacterial wilt, *Ralstonia solanacearum*, rhizosphere microbiome, potential biocontrol agents, dysbiosis, metagenomics

## Abstract

Tobacco bacterial wilt, caused by *Ralstonia solanacearum*, threatens global tobacco production. While the rhizosphere microbiome defends against soil-borne pathogens, mechanisms underlying how bacterial wilt reshapes microbial community structure, function, and ecological interactions remain poorly understood. Here, we employed metagenomic sequencing to investigate taxonomic and functional alterations in the rhizosphere microbiome of symptomatic (S) and asymptomatic (A) tobacco plants across two locations (Fenggang and Bozhou), establishing four groups: FA, FS, BA, and BS. Quality control of sequencing data showed no technical bias between groups (*p* > 0.05). Contrary to the paradigm that pathogen invasion reduced microbial diversity, alpha diversity analysis revealed higher species richness (Sobs) in symptomatic soils, whereas community evenness (Shannon and Simpson indices) remained unchanged, suggesting selective reshuffling rather than microbiome collapse. Beta-diversity analysis revealed significant compositional shifts associated with disease status (PERMANOVA, *R*^2^ = 0.713, *p* = 0.001), with symptomatic communities displaying greater heterogeneity. Taxonomic profiling revealed consistent enrichment of the pathogen *R. solanacearum* and opportunistic bacteria (including *Stenotrophomonas* and *Pseudomonas*) in symptomatic rhizospheres, concomitant with depletion of putative beneficial taxa (Candidatus_Solibacter, *Luteitalea*, and *Metarhizium*). Functional annotation indicated a metabolic shift from homeostatic maintenance to stress adaptation and pathogenicity. Symptomatic soils exhibited significant enrichment of virulence factors, including motility and secretion system genes, microbial defense mechanism genes (COG), and antibiotic resistance genes (CARD). Additionally, increased abundance of carbohydrate-active enzymes (CAZy)—particularly glycoside hydrolases—suggested intensive nutrient acquisition from decaying tissues. Co-occurrence network analysis revealed that asymptomatic communities formed denser, competition-driven networks characterized by a higher proportion of negative correlations. Disease destabilized these networks by reducing connectivity and, crucially, rewired interactions of *R. solanacearum* from negative to positive associations with taxa such as *Sphingobium*, thereby reflecting erosion of competitive constraints and pathogen incorporation into cooperative networks. Our findings revealed that bacterial wilt drove multi-layered dysbiosis, encompassing pathogen-driven taxonomic selection, functional shifts toward stress adaptation and intensified competition, and collapse of stable antagonistic networks associated with plant health. This study provided mechanistic insights into microbiome-mediated disease progression and identified specific microbial taxa and network properties as candidate targets for ecological disease management and early diagnostic indicators.

## 1 Introduction

Plant health is fundamental to agricultural productivity and global food security. However, soilborne diseases constrain crop yields worldwide, causing annual economic losses that exceed billions of dollars (Panth et al., 2020). The rhizosphere microbiome—a diverse microbial consortium colonizing the zone surrounding plant roots—reaches densities of up to 10^11^ cells per gram of root and harbors more than 3,000 prokaryotic species, serving as a critical interface for plant-soil interactions (Egamberdieva et al., 2008; Mendes et al., 2011). Often regarded as the plant’s “second genome,” the rhizosphere microbiome plays essential roles in nutrient acquisition, stress tolerance, and pathogen suppression (Berendsen et al., 2012). Understanding the compositional and functional dynamics of this complex ecosystem is therefore essential for developing sustainable disease management strategies.

Bacterial wilt, caused by the soil-borne pathogen *Ralstonia solanacearum* species complex (RSSC), ranks among the most devastating vascular diseases affecting Solanaceae crops, including tobacco (*Nicotiana tabacum*), potato, tomato, and pepper (Ahmed et al., 2022; Liu et al., 2024). In tobacco cultivation, bacterial wilt exhibits spatiotemporal variability, with baseline prevalence of 15–35% that surges beyond 75% when co-occurring with black shank, eroding 50–60% of yields in wet mono-cropping systems and reaching 100% losses during extreme outbreaks (Jiang et al., 2017). No commercial tobacco cultivars exhibit durable resistance to RSSC despite decades of breeding efforts. Current management paradigms predominantly rely on cultural practices, crop rotation, and prophylactic pesticide applications (Lee et al., 2012; Meline et al., 2023). These approaches remain insufficient under field conditions, however. Furthermore, the indiscriminate application of chemical pesticides has detrimental consequences, including soil microbiome disruption, environmental contamination, and selection for resistant pathogen populations (Dangi et al., 2017; Verdadero et al., 2025). This impasse necessitates a paradigm shift toward harnessing indigenous beneficial microorganisms as a cornerstone of next-generation disease control strategies.

The concept of disease-suppressive soils provides compelling evidence for the potential of microbiome-mediated pathogen control (De Corato, 2020). These soils exhibit an intrinsic capacity to restrain pathogen establishment or proliferation despite the presence of a susceptible host and conducive environmental conditions (Mendes et al., 2011; Schlatter et al., 2017). This suppressive phenotype emerges from the collective antagonistic activities of the resident microbial community rather than individual microbial taxa. During plant development, root exudates selectively recruit specific microbial cohorts from bulk soil to the rhizosphere and endosphere (Hayden et al., 2019; Raaijmakers et al., 2009). These recruited beneficial microorganisms suppress pathogens through multiple mechanisms, including direct antagonism via antimicrobial compound production, competition for nutrients and niche occupation, induction of systemic resistance, and modulation of root exudate profiles (Bonanomi et al., 2018; Liu et al., 2024). Numerous studies have consistently identified key bacterial genera—including *Pseudomonas*, *Streptomyces*, *Bacillus*, and *Paenibacillus*—as primary contributors to disease suppression in various agricultural systems (Carrión et al., 2019; Peng et al., 2025; Raaijmakers and Mazzola, 2016).

Despite the recognized potential of indigenous microbiota in disease suppression, the mechanisms through which *R. solanacearum* subverts these protective interactions during infection remain poorly characterized. As a hemibiotrophic vascular pathogen, *R. solanacearum* employs a multi-stage infection process involving root surface colonization, tissue invasion, and systemic proliferation within xylem vessels (Genin and Denny, 2012). This pathogenic progression inevitably disrupts the rhizosphere microbiome. Yet the specific taxa displaced, the functional gene networks compromised, and the temporal dynamics of this community destabilization have not been thoroughly characterized. Although microbiome dysbiosis—defined as compositional and functional changes that disrupt microbial community stability and disease-suppressive capacity—has been documented in other plant-pathogen systems (Levy et al., 2018; Stappenbeck and Virgin, 2016), the distinctive features of RSSC infection suggest distinct patterns of rhizosphere disruption in tobacco. Importantly, the keystone microbial species that maintain disease-suppressive functions, the metabolic pathways associated with pathogen resistance, and the early-warning indicators of microbiome destabilization have not been systematically investigated in tobacco agroecosystems.

To address these knowledge gaps, we employed metagenomic sequencing to investigate compositional and functional shifts in the tobacco rhizosphere microbiome during bacterial wilt development. Specifically, we assessed rhizosphere dysbiosis using quantitative indicators across multiple dimensions: shifts in alpha diversity; enrichment of potential pathogenic taxa and depletion of beneficial genera; alterations in microbial co-occurrence network properties; and changes in functional gene profiles. By comparing microbiome profiles across symptomatic and asymptomatic rhizosphere soils, we aim to (1) elucidate disease-induced changes in microbial diversity and community composition; (2) characterize shifts in community functional potential, particularly in pathways related to nutrient cycling and pathogen suppression; and (3) construct co-occurrence networks to reveal disrupted microbial interactions. This study provides an integrated perspective on the structural and functional dimensions of rhizosphere dysbiosis associated with bacterial wilt, establishing a foundation for future microbiome-based strategies to manage this devastating disease.

## 2 Materials and methods

### 2.1 Sample collection

**FIGURE 1:**
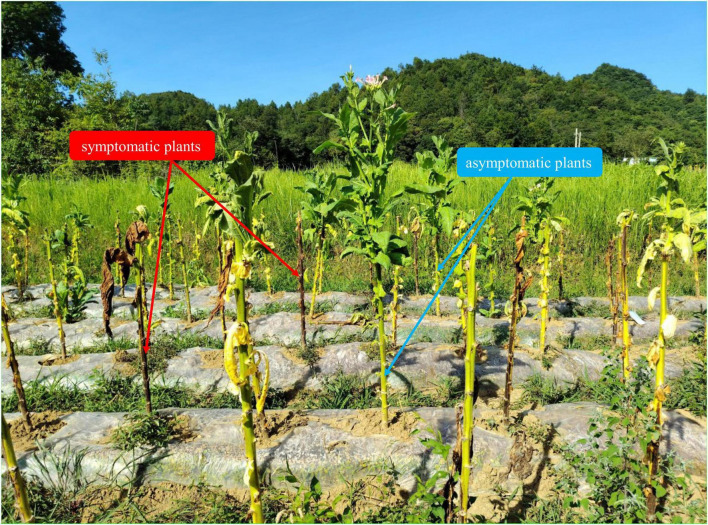
Representative field photographs of symptomatic and asymptomatic tobacco plants at sampling.

Field sampling was conducted in August 2025 at two major tob acco-producing counties in Zunyi City, Guizhou Province, China: Fenggang (F; 107°40′48″E, 27°47′45″N) and Bozhou (B; 106°39′58″E, 27°38′34″N). The tobacco cultivar was “Yunyan 87.” At each location, rhizosphere soil was collected from five symptomatic (S, plants exhibiting clear bacterial wilt symptoms) and five asymptomatic (A) plants (Figure 1). Rhizosphere soil, defined as soil tightly adhering to roots, was collected following an established protocol (Bulgarelli et al., 2012). Briefly, after gently removing loosely attached bulk soil, rhizosphere soil was obtained by brushing the root surface with a sterile brush. This sampling design yielded four experimental groups (FS, FA, BS, and BA), with a total of 20 independent biological replicates. All samples were immediately placed on dry ice in the field, transported to the laboratory, and stored at –80°C until DNA extraction.

### 2.2 DNA extraction, library preparation, and metagenomic sequencing

Total genomic DNA was extracted from 0.2 g of each frozen rhizosphere soil sample using the E.Z.N.A.^®^ soil DNA Kit (Omega Bio-tek, Norcross, GA, United States) following the manufacturer’s protocol. DNA integrity was assessed by 1% agarose gel electrophoresis, and DNA concentration and purity were quantified using a NanoDrop-2000 spectrophotometer (Thermo Fisher Scientific, Waltham, MA, United States). Only samples with an A260/A280 ratio between 1.8 and 2.0 and no evident degradation were retained for downstream analysis.

Qualified genomic DNA was sheared to an average size of ∼350 bp using a Covaris M220 focused-ultrasonicator (Covaris, Woburn, MA, United States). Sequencing libraries were constructed using the NEXTFLEX Rapid DNA-Seq kit (Bioo Scientific, Austin, TX, United States) following the standard Illumina paired-end (PE) protocol. The workflow comprised (1) end repair and A-tailing, (2) ligation of Illumina PE adapters, (3) purification of adapter-ligated fragments using magnetic beads to remove self-ligated adapter dimers, and (4) limited-cycle PCR amplification to enrich library templates. The completed libraries were denatured with sodium hydroxide to generate single-stranded DNA. Bridge amplification and cluster generation were performed on an Illumina flow cell. Finally, sequencing was performed on an Illumina NovaSeq™ X Plus (Illumina Inc., San Diego, CA, United States) at Majorbio Bio-Pharm Technology Co., Ltd. (Shanghai, China) using the NovaSeq X Series 25B Reagent Kit according to the manufacturer’s instructions.^1^ The raw metagenomic sequencing data have been deposited in the NCBI Sequence Read Archive under accession number PRJNA1403154.

### 2.3 Data processing and taxonomic and functional annotation

All bioinformatics analyses were performed on the Majorbio Cloud Platform.^2^ Briefly, raw sequencing reads were first processed by fastp (version 0.20.0)^3^ to remove adapter sequences and low-quality reads (length < 50 bp or average Phred quality value < 20). Quality-filtered reads were subsequently assembled into contigs using MEGAHIT (version 1.2.9),^4^ and only contigs ≥ 300 bp were retained for downstream analysis (Li et al., 2015). Open reading frames (ORFs) were predicted from assembled contigs using Prodigal (version 2.6.3),^5^ and ORFs shorter than 100 bp were discarded (Hyatt et al., 2010). A non-redundant gene catalog was constructed using CD-HIT (version 4.7)^6^ with thresholds of 90% sequence identity and 90% coverage (Fu et al., 2012). Gene abundance in each sample was quantified using SOAPaligner (version 2.21)^7^ with a minimum alignment identity of 95% (Li et al., 2008).

For taxonomic annotation, non-redundant genes were aligned against the NCBI NR database using DIAMOND (version 2.0.13)^8^ with an *e*-value cutoff of 1e-5 (Buchfink et al., 2015). For functional annotation, sequences were annotated against several databases, including the Kyoto Encyclopedia of Genes and Genomes (KEGG), Gene Ontology (GO), Clusters of Orthologous Groups (COG), Carbohydrate-Active Enzymes (CAZy), Comprehensive Antibiotic Resistance Database (CARD), and Pathogen-Host Interactions (PHI) database. Differential abundance analysis across taxonomic, functional, and gene levels was performed using Kruskal–Wallis tests (for multiple group comparisons) and Wilcoxon rank-sum tests (for two-group comparisons), based on annotated profiles and non-redundant gene abundance tables. Dunn’s *post-hoc* test was applied for pairwise comparisons. All *p*-values from multiple comparisons were adjusted using the Benjamini–Hochberg method to control for false discovery rate (FDR), and adjusted *p* < 0.05 was considered statistically significant.

### 2.4 Data analysis

Unless otherwise stated, all statistical analyses were conducted in R (version 3.6.3). Gene richness and alpha diversity indices were calculated for each sample using custom R scripts based on the vegan package (version 2.5–7).^9^ Bray-Curtis dissimilarity matrices were calculated from gene abundance profiles to assess beta diversity. Principal coordinate analysis (PCoA) was performed using Bray-Curtis distance to visualize compositional differences among samples based on genus-level taxonomic annotations, with results plotted using ggplot2 (Ginestet, 2011). Permutational multivariate analysis of variance (PERMANOVA) was employed to evaluate effects of sampling location and disease status on community composition, explanatory power was quantified using *R*^2^ with significance determined by 999 permutations (Anderson, 2001). Heatmaps were generated using Pheatmap (version 1.0.12) to visualize variation in relative abundances of key microbial genera. Hierarchical clustering was performed based on Euclidean distance of log-transformed gene abundance profiles. Co-occurrence networks were constructed for the top 50 abundant genera based on Spearman’s rank correlations. To ensure robust and biologically meaningful associations, only correlations with |r| > 0.7 were retained as edges, a threshold widely used in microbial network studies (Barberán et al., 2012; de Menezes et al., 2015) to focus on moderate to strong correlations and minimize spurious associations. All *p*-values were adjusted using the Benjamini–Hochberg FDR method, and only correlations with FDR-adjusted *p* < 0.05 were considered statistically significant. Network visualization was performed using Cytoscape with the CoNet plugin (Faust and Raes, 2016), and subsequent topological analysis was conducted using Gephi (version 0.9.2; Jacomy et al., 2014).

## 3 Results

### 3.1 Sequencing data overview and quality assessment

Metagenomic sequencing of samples from the four treatment groups (FA, FS, BA, and BS) generated a comprehensive dataset for microbial community characterization. Following quality control, including adapter trimming and quality filtering, a total of 860.9 million clean reads were retained from 871.1 million raw reads, representing a 98.7% retention rate (Table 1). Sample-wise *de novo* assembly produced 110,607–245,615 contigs per sample, with cumulative assembly lengths ranging from 75.9 and 209.1 Mb (Table 1). The assemblies exhibited high continuity, with a mean N50 value of 751 ± 54 bp across all samples. Gene prediction identified 4,632,122 putative ORFs in total, with an average of 231,606 ± 50,420 ORFs per assembly (Table 2). The mean ORF length was 499 ± 19 bp. Notably, comparative assessments within each sampling site revealed no statistically significant differences (*p* > 0.05) in sequencing depth or key assembly parameters (i.e., total contig length and N50 values) between asymptomatic and symptomatic groups. These results confirmed that subsequent comparative analyses were not confounded by technical artifacts associated with disease status.

**TABLE 1:** Metagenomic sequencing and assembly statistics for rhizosphere soil samples.

Samples	Raw reads (Million)	Clean reads (Million)	Number of contigs	Total contig length (Mb)	N50 (bp)
FA1	42.6	42.2	190,153	155.4	777
FA2	46.9	46.3	201,333	182.0	850
FA3	40.0	39.6	151,490	120.8	751
FA4	42.7	42.2	152,211	121.9	755
FA5	41.5	41.0	178,573	146.3	784
FA Mean ± SD	42.7 ± 2.6a	42.3 ± 2.5a	174,752 ± 22,403a	145.3 ± 25.5a	783 ± 40a
FS1	44.6	44.1	198,548	161.1	771
FS2	49.8	49.2	245,615	209.1	813
FS3	39.6	39.2	152,052	127.1	782
FS4	40.0	39.6	161,864	130.6	759
FS5	40.1	39.7	143,653	114.4	749
FS mean ± SD	42.8 ± 4.4a	42.4 ± 4.3a	180,346 ± 42,071a	148.5 ± 38.0a	775 ± 25a
BA1	39.8	39.3	129,340	97.8	714
BA2	47.3	46.8	153,982	115.4	704
BA3	48.2	47.7	164,621	121.8	698
BA4	48.1	47.4	186,628	144.9	740
BA5	40.7	40.3	110,607	75.9	653
BA Mean ± SD	44.8 ± 4.2a	44.3 ± 4.1a	149,036 ± 29,770a	111.2 ± 25.9a	702 ± 32a
BS1	46.3	45.7	153,669	115.7	700
BS2	42.8	42.3	124,034	120.7	866
BS3	45.2	44.4	143,881	107.4	701
BS4	41.8	41.3	135,669	98.5	687
BS5	43.1	42.6	184,846	145.7	756
BS mean ± SD	43.8 ± 1.9a	43.3 ± 1.8a	148,420 ± 23,088a	117.6 ± 17.8a	742 ± 74a
Grand mean ± SD	43.6 ± 3.3	43.0 ± 3.2	163,138 ± 31,621	130.6 ± 30.6	751 ± 54

Statistical comparisons were performed using the Kruskal-Wallis test followed by Dunn’s *post hoc* test with Benjamini-Hochberg FDR correction. No significant differences were detected among groups (FDR-adjusted *p* > 0.05). FA, Fenggang asymptomatic soils; FS, Fenggang symptomatic soils; BA, Bozhou asymptomatic soils; BS, Bozhou symptomatic soils.

**TABLE 2:** Statistics of open reading frame (ORF) prediction and gene catalog construction.

Sample	No. of ORFs	Total ORF length (Mb)	Average ORF length (bp)
FA1	273,503	138.7	507
FA2	303,565	160.8	530
FA3	213,863	107.7	503
FA4	216,907	109.2	503
FA5	257,705	130.5	507
FA mean ± SD	253,109 ± 38,189a	129.4 ± 22.1a	510 ± 11a
FS1	287,979	144.1	501
FS2	362,467	186.9	516
FS3	221,914	113.5	511
FS4	233,244	116.8	501
FS5	204,402	102.7	502
FS mean ± SD	262,001 ± 64,265a	132.8 ± 33.9a	506 ± 7a
BA1	175,872	85.8	488
BA2	205,056	98.8	482
BA3	221,422	106.5	481
BA4	261,344	128.9	493
BA5	143,243	66.9	467
BA mean ± SD	201,387 ± 44,837a	97.4 ± 23.1a	482 ± 10a
BS1	211,997	102.6	484
BS2	194,498	107.4	552
BS3	196,940	94.9	482
BS4	182,472	87.1	478
BS5	263,729	130.6	495
BS mean ± SD	209,927 ± 31,857a	104.5 ± 16.5a	498 ± 31a
Grand mean ± SD	231,606 ± 50,420	116.0 ± 27.6	499 ± 19

Statistical significance was assessed using the Kruskal-Wallis test with Benjamini-Hochberg FDR correction for multiple comparisons. No significant differences were detected among groups for any parameter (FDR-adjusted *p* > 0.05), confirming comparable gene prediction and catalog construction quality across samples. FA, Fenggang asymptomatic soils; FS, Fenggang symptomatic soils; BA, Bozhou asymptomatic soils; BS, Bozhou symptomatic soils.

### 3.2 Structural shifts in the rhizosphere microbial community

#### 3.2.1 Disease-induced changes in alpha and beta diversity

Alpha diversity analysis revealed significant differences in species richness among groups (Table 3). At both sampling sites, asymptomatic groups exhibited lower observed species numbers (Sobs) than symptomatic groups. *Post-hoc* comparisons with Benjamini-Hochberg FDR correction confirmed a significant difference at Bozhou but not at Fenggang. Specifically, the BA group had the lowest mean species richness (17,644 ± 582), which differed significantly from the other three groups (FDR-adjusted *p* < 0.05 for all pairwise comparisons). Conversely, other alpha diversity indices, including Shannon, Simpson, and Pielou, showed no statistically significant differences among the four groups (Kruskal-Wallis test, *p* > 0.05), suggesting that disease status did not substantially alter community evenness.

**TABLE 3:** Alpha diversity indices of rhizosphere microbial communities across four experimental groups.

Sample	Sobs	Shannon	Simpson	Pielou_e
FA1	19,507	6.10	0.011	0.62
FA2	19,320	6.03	0.018	0.61
FA3	18,610	5.99	0.012	0.61
FA4	19,385	6.01	0.014	0.61
FA5	19,515	6.13	0.011	0.62
FA mean ± SD	19,267 ± 377ab	6.05 ± 0.06a	0.013 ± 0.003a	0.61 ± 0.01a
FS1	19,943	6.13	0.013	0.62
FS2	20,719	6.16	0.010	0.62
FS3	19,663	6.18	0.012	0.62
FS4	19,774	6.16	0.012	0.62
FS5	19,125	6.04	0.014	0.61
FS mean ± SD	19,844 ± 577a	6.13 ± 0.06a	0.012 ± 0.002a	0.62 ± 0.00a
BA1	17,147	5.83	0.013	0.60
BA2	17,886	5.93	0.012	0.61
BA3	18,119	5.93	0.011	0.60
BA4	18,185	5.97	0.011	0.61
BA5	16,884	5.85	0.014	0.60
BA mean ± SD	17,644 ± 582c	5.90 ± 0.06a	0.012 ± 0.001a	0.60 ± 0.01a
BS1	18,753	6.08	0.010	0.62
BS2	18,504	5.32	0.025	0.54
BS3	18,549	6.07	0.010	0.62
BS4	18,339	5.92	0.011	0.60
BS5	18,531	5.80	0.013	0.59
BS mean ± SD	18,535 ± 148b	5.84 ± 0.31a	0.014 ± 0.006a	0.59 ± 0.03a
Grand mean ± SD	18,823 ± 945	5.98 ± 0.19	0.013 ± 0.003	0.61 ± 0.02

Statistical comparisons were performed using the Kruskal-Wallis test followed by Dunn’s *post-hoc* test with Benjamini-Hochberg FDR correction. Different lowercase letters indicate significant differences (FDR-adjusted *p* < 0.05). Sobs, observed species richness; Shannon, Shannon-Wiener diversity index; Simpson, Simpson diversity index; Pielou_e, Pielou’s evenness index. FA, Fenggang asymptomatic soils; FS, Fenggang symptomatic soils; BA, Bozhou asymptomatic soils; BS, Bozhou symptomatic soils.

**FIGURE 2:**
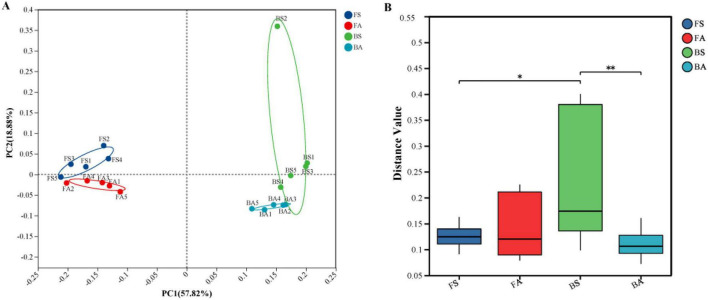
Beta diversity patterns and compositional dissimilarity of rhizosphere microbial communities across four experimental groups. **(A)** Principal coordinate analysis (PCoA) based on Bray-Curtis dissimilarities of genus-level community composition. Percentage of variance explained by each axis is indicated in parentheses. **(B)** Box plots showing pairwise Bray-Curtis dissimilarities within and between groups. Significance was determined by permutational multivariate analysis of variance (PERMANOVA; *R*^2^ = 0.713, *p* = 0.001). Pairwise comparisons were performed using Wilcoxon rank-sum tests with Benjamini–Hochberg FDR correction. * FDR-adjusted *p* < 0.05, ** FDR-adjusted *p* < 0.01. FA, Fenggang asymptomatic soils; FS, Fenggang symptomatic soils; BA, Bozhou asymptomatic soils; BS, Bozhou symptomatic soils.

Principal coordinate analysis (PCoA) revealed clear separation among groups along the first two axes (Figure 2A), which accounted for 57.82 and 18.88% of the total variance on PC1 and PC2, respectively. Samples from the same sampling site clustered more closely than those from different sites, regardless of disease status. Permutational multivariate analysis of variance (PERMANOVA) confirmed that these compositional differences were statistically significant (*R*^2^ = 0.713, *p* = 0.001). Analysis of Bray-Curtis dissimilarities (Figure 2B), revealed that the median dissimilarity of group BS exceeded that of other groups, with its distance values differing significantly from those of groups FS and BA.

#### 3.2.2 Disease-induced taxonomic alterations across multiple levels

**FIGURE 3:**
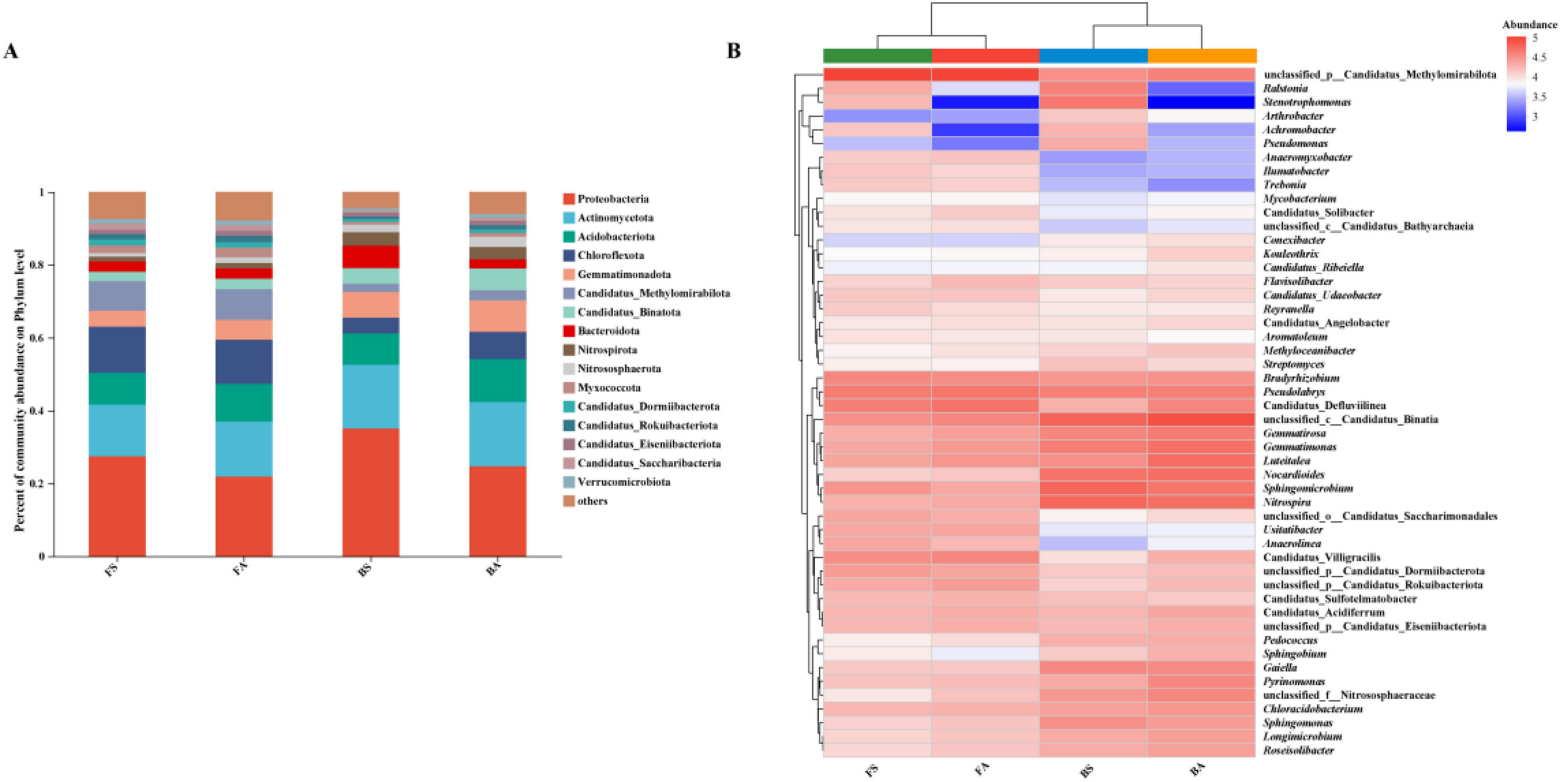
Taxonomic composition and relative abundance of rhizosphere microbial communities at phylum and genus levels. **(A)** Relative abundance of dominant bacterial phyla across four experimental groups. Taxa with relative abundance < 1% were classified as “Others.” **(B)** Heatmap showing relative abundance of the top 50 bacterial genera, with hierarchical clustering based on Euclidean distance of log-transformed abundance data. Color scale indicates relative abundance. FA, Fenggang asymptomatic soils; FS, Fenggang symptomatic soils; BA, Bozhou asymptomatic soils; BS, Bozhou symptomatic soils.

At the phylum level, the microbial community was predominantly composed of Proteobacteria, Actinomycetota, Acidobacteriota, Chloroflexota, Gemmatimonadota, and Bacteroidota (Figure 3A). Tobacco bacterial wilt induced significant structural alterations in this community. Specifically, at Fenggang, the relative abundance of Proteobacteria increased significantly, while that of Acidobacteriota decreased substantially. Similarly, at Bozhou, Proteobacteria and Bacteroidota were significantly enriched, whereas Acidobacteriota and Chloroflexota were notably depleted. To identify specific microbial genera associated with plant health status, a heatmap analysis was performed at the genus level (Figure 3B). *Ralstonia* (Proteobacteria) the causal agent of tobacco bacterial wilt, was markedly enriched in symptomatic samples (FS and BS). Genera such as *Stenotrophomonas* and *Achromobacter* (both Proteobacteria) also exhibited consistently higher relative abundance in the symptomatic groups compared to asymptomatic counterparts. Several other genera were widely distributed across all samples, including *Bradyrhizobium*, *Pseudolabrys*, *Sphingomicrobium*, and *Sphingomonas* (Proteobacteria); *Gemmatirosa*, *Gemmatimonas*, and *Longimicrobium* (Gemmatimonadota); *Nocardioides*, *Gaiella*, *Pyrinomonas*, and *Roseisolibacter* (Actinomycetota); and *Luteitalea* (Verrucomicrobiota), *Nitrospira* (Nitrospirota), and *Chloracidobacterium* (Acidobacteriota).

**FIGURE 4:**
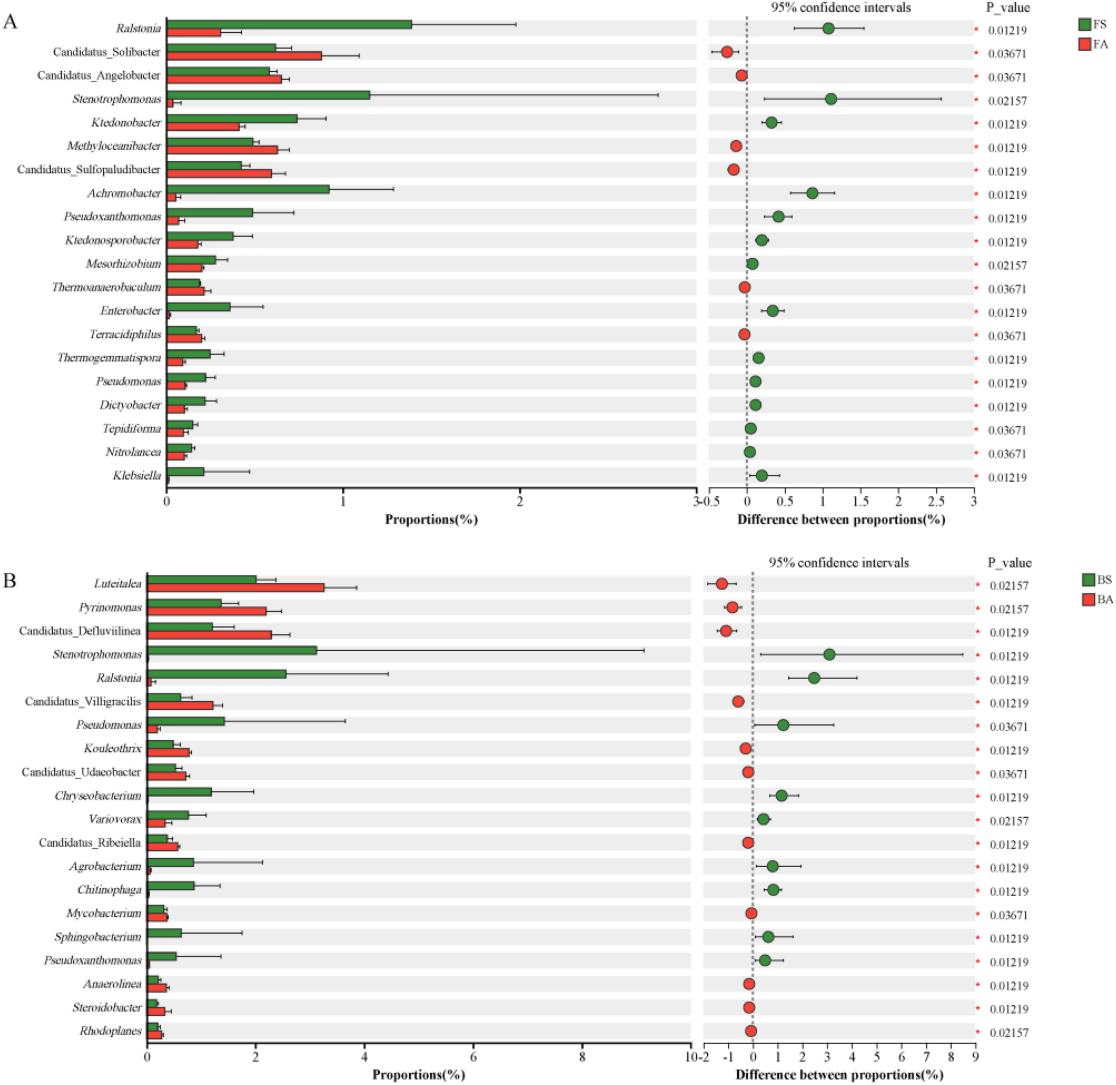
Differentially abundant bacterial genera between symptomatic and asymptomatic rhizosphere soils identified by Wilcoxon rank-sum tests. **(A)** Fenggang. **(B)** Bozhou. Bars represent the top 20 most abundant genera exhibiting significant differences between groups. Significance was determined by Wilcoxon rank-sum tests with Benjamini–Hochberg FDR correction. Only genera with FDR-adjusted *p* < 0.05 are shown. Green bars indicate enrichment in symptomatic soils, red bars indicate enrichment in asymptomatic soils.

To further identify bacterial genera exhibiting significant shifts in response to tobacco bacterial wilt at each location, Wilcoxon rank-sum tests were performed on genus-level compositional data (top 20 most abundant taxe) (Figure 4). At Bozhou, symptomatic soils exhibited marked enrichment of the pathogen *Ralstonia*, along with *Stenotrophomonas*, *Pseudomonas*, *Chryseobacterium*, and *Agrobacterium* (*p* < 0.05). Conversely, *Luteitalea*, *Pyrinomonas*, *Kouleothrix*, *Methyloceanibacter*, Candidatus_Defluviilinea, Candidatus_Villigracilis, Candidatus_Sulfopaludibacter, and Candidatus_Solibacter were significantly elevated in asymptomatic soils. A comparable pattern was observed at Fenggang, where *Ralstonia* again was the most significantly enriched genus in symptomatic samples. *Achromobacter*, *Enterobacter*, and *Klebsiella* also demonstrated substantial increases in symptomatic soils, whereas *Methyloceanibacter*, *Terracidiphilus*, *Thermoanaerobaculum*, Candidatus_Solibacter, Candidatus_Angelobacter, and Candidatus_Sulfopaludibacter predominated in asymptomatic soils. Despite site-specific variations, *Ralstonia* remained the most consistently enriched genus across symptomatic samples, supporting its pivotal role in disease progression. *Stenotrophomonas* and *Pseudomonas* also showed repeatable enrichment in symptomatic soils, indicating their potential as microbial indicators of tobacco bacterial wilt.

**FIGURE 5:**
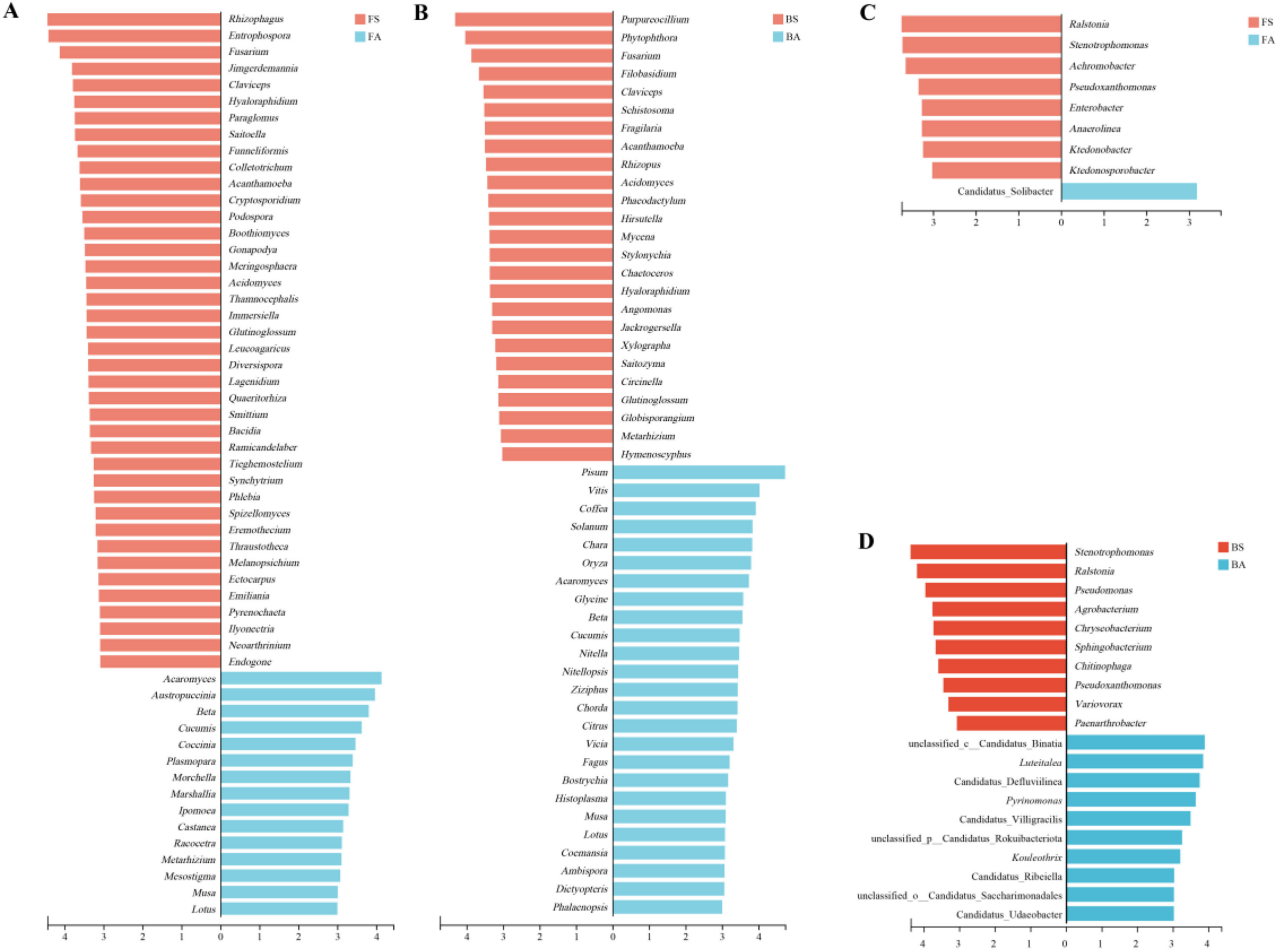
LEfSe-identified biomarker taxa distinguishing symptomatic and asymptomatic rhizosphere microbiomes. Linear discriminant analysis effect size (LEfSe) biomarker analysis (LDA > 3, *p* < 0.05) identifying discriminative fungal and bacterial genera between symptomatic and asymptomatic rhizosphere soils at Fenggang **(A,C)** and Bozhou **(B,D)**. **(A)** Fungal biomarkers at Fenggang; **(B)** Fungal biomarkers at Bozhou; **(C)** Bacterial biomarkers at Fenggang; **(D)** Bacterial biomarkers at Bozhou. Red bars indicate enrichment in symptomatic soils; blue bars indicate enrichment in asymptomatic soils.

Linear discriminant analysis Effect Size (LEfSe) analysis (LDA > 3, *p* < 0.05) was performed to identify key taxa in symptomatic and asymptomatic samples (Figure 5). This analysis identified 55 eukaryotic and 9 bacterial genera as biomarkers at Fenggang, and 50 eukaryotic and 20 bacterial genera at Bozhou. Eukaryotic biomarker genera consistently associated with symptomatic samples at both locations included *Fusarium*, *Claviceps*, *Hyaloraphidium*, *Acanthamoeba*, *Acidomyces*, and *Glutinoglossum*. Bacterial biomarker common to both sites were *Ralstonia*, *Stenotrophomonas*, and *Pseudoxanthomonas*. In the asymptomatic group, fungal biomarkers (excluding plant and algal biomarkers) comprised *Metarhizium*, *Racocetra*, *Morchella*, *Austropuccinia*, *Acaromyces*, *Ambispora*, *Coemansia*, and *Histoplasma*, whereas bacterial biomarkers comprised Candidatus_Solibacter, *Luteitalea*, *Pyrinomonas*, and *Kouleothrix*.

### 3.3 Functional characterization of the rhizosphere microbiome

**FIGURE 6:**
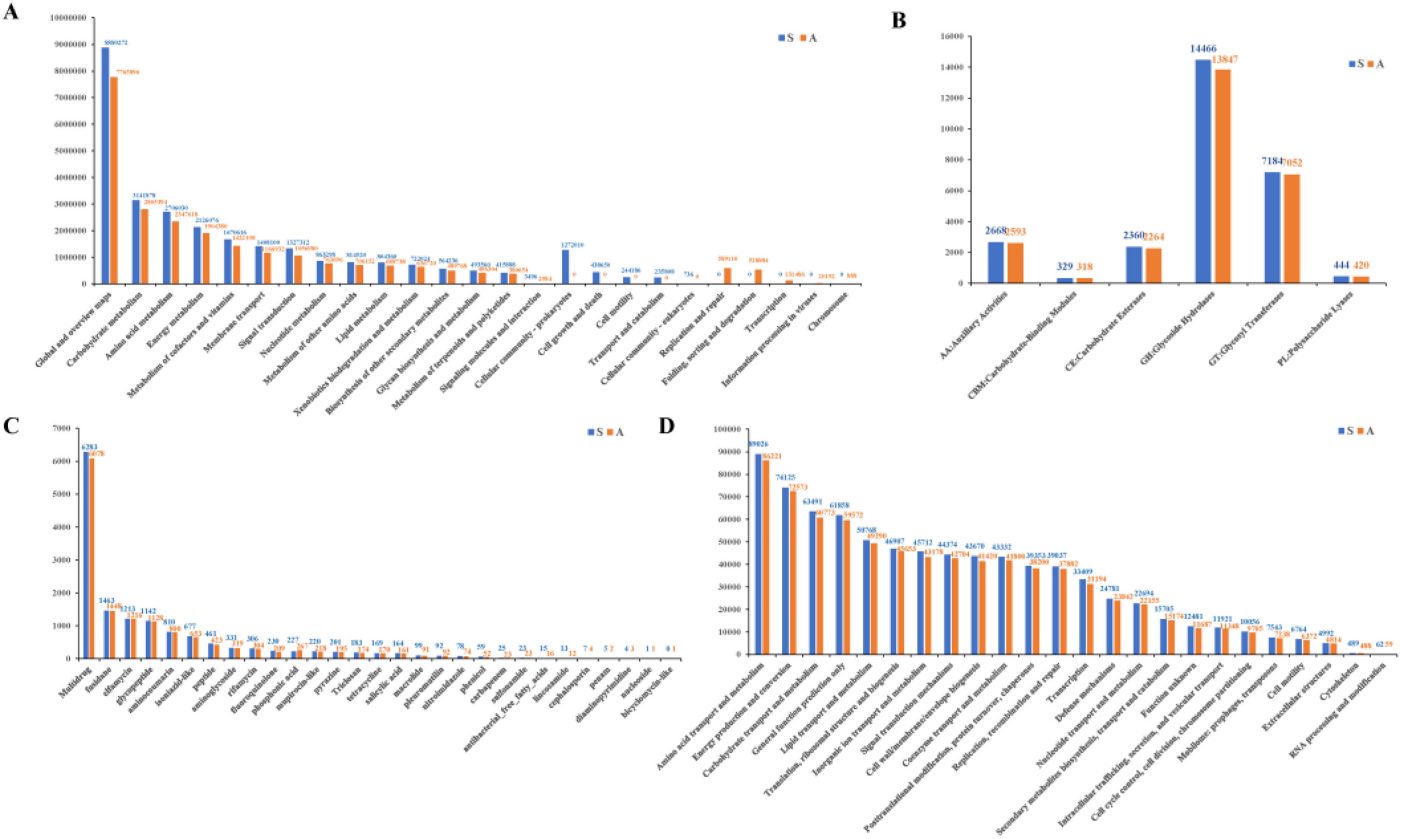
Functional reprograming of rhizosphere microbiomes in response to tobacco bacterial wilt. **(A)** KEGG functional pathway enrichment. **(B)** Carbohydrate-active enzyme (CAZy) profiles. **(C)** Antibiotic resistance gene (CARD) categories. **(D)** COG functional classification. Blue bars indicate enrichment in symptomatic soils. Orange bars indicate enrichment in asymptomatic soils.

Comparative analysis of rhizosphere microbiome functional potential revealed distinct metabolic alterations associated with tobacco bacterial wilt. KEGG Orthology (KO) annotation identified a substantial functional shift, with symptomatic rhizosphere microbiome exhibiting increased representation across most functional categories. Specifically, genes involved in global and overview maps were markedly enriched in symptomatic samples (8,880,272 vs. 7,765,896 KO assignments in asymptomatic soils) (Figure 6A). Additionally, functional modules related to cellular community-prokaryotes, cell growth and death, cell motility, and transport and catabolism were detected exclusively in symptomatic samples. In contrast, pathways associated with replication and repair, protein processing (folding, sorting and degradation), transcription, information processing in viruses, and chromosome maintenance predominated in asymptomatic soils. CAZy database analysis revealed a broad-spectrum increase across carbohydrate-active enzymes, particularly glycoside hydrolases (GHs), with higher abundance in symptomatic (14,466) than in asymptomatic (13,847) groups (Figure 6B). This suggests enhanced potential for plant-derived carbohydrate degradation, potentially facilitating nutrient acquisition from stressed root tissues and contributing to plant cell wall degradation.

Analysis of antibiotic resistance genes using the Comprehensive Antibiotic Resistance Database (CARD) revealed a distinct resistance profile associated with disease. Specifically, 27 resistance categories showed elevated abundances in symptomatic soils, whereas only two categories were higher in asymptomatic soils (Figure 6C). This pattern contrasts with typical observations, where resistance genes generally predominate in asymptomatic rhizospheres. The enrichment of defense-related functions in symptomatic soils suggests a community-wide stress response, potentially driven by pathogen-induced immune activation or inter-microbial competition. COG functional classification further supported this stress-response phenotype: symptomatic group exhibited increased abundance of genes associated with defense mechanisms (Figure 6D), whereas core metabolic categories such as amino acid transport and metabolism were relatively depleted.

### 3.4 Microbial interaction networks under different statuses

**FIGURE 7:**
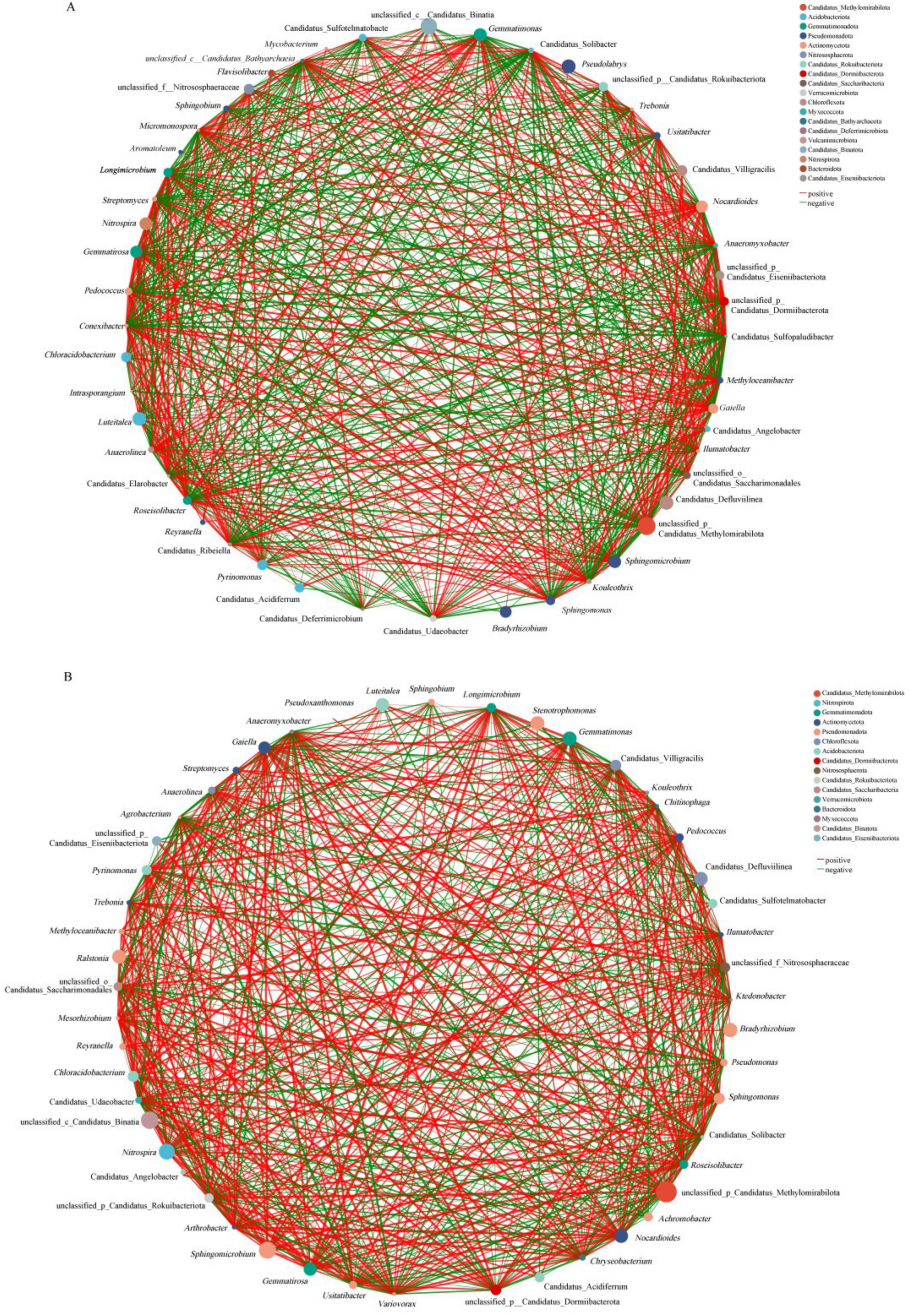
Co-occurrence networks of dominant bacterial genera in asymptomatic **(A)** and symptomatic **(B)** rhizosphere soils. Networks were constructed based on Spearman’s rank correlations (| *r*| > 0.7, *p* < 0.05) among the top 50 most abundant microbial genera. Node size is proportional to relative abundance; edge thickness reflects the absolute correlation coefficient magnitude. Red edges, positive correlations; green edges, negative correlations. Node degree (number of connections) indicates interaction frequency within the community.

To elucidate the impact of tobacco bacterial wilt on interspecies relationships within the rhizosphere microbiome, co-occurrence networks were constructed separately for asymptomatic (Figure 7A) and symptomatic (Figure 7B) groups. Networks were inferred based on robust Spearman correlations between relative abundances of microbial taxa, with significant edges (| *r*| > 0.7, *p* < 0.05) retained to ensure ecological relevance and comparability. Compared with the symptomatic network (604 edges: including 270 negative and 334 positive correlations), the asymptomatic network exhibited more connections (665 edges), and a higher proportion of negative correlations (329 vs. 270 in the symptomatic group). These findings indicate that microbial interactions in asymptomatic soils were not only more frequent but also more antagonistic, suggesting stronger mutual constraints among taxa. Such competitive environments may suppress pathogen proliferation, thereby contributing to plant health maintenance and reduced disease incidence.

Specifically, several genera exhibited marked shifts in network importance. *Nitrospira* and Candidatus_Villigracilis, for example, showed substantially reduced connectivity in the asymptomatic network compared to the symptomatic network (Supplementary Table S1), suggesting suppression of nitrification-related taxa under healthy conditions. In contrast, *Sphingobium* and *Methyloceanibacter* were significantly more central in the asymptomatic network, indicating enhanced roles in organic compound degradation and stress response, that may contribute to community stability and pathogen exclusion. The asymptomatic network uniquely contained several genera with well-established beneficial functions, including *Pseudomonas*, *Arthrobacter*, and *Variovorax*, which are characterized by direct antagonism, resource competition, and plant growth promotion, thereby forming a disease-suppressive consortium. The absence of *Ralstonia* from this network exhibited confirms its successful suppression. Conversely, the symptomatic network exhibited emergence of new, highly connected core genera, including *Streptomyces* and *Micromonospora*—prolific antibiotic producers—suggesting that a stressed community formed alternative defensive alliances. This node-level analysis revealed that bacterial wilt not only reduced overall microbial interactions but also drove functional restructuring of the network core, replacing a beneficial, competition-driven hub structure with a reconfigured state facilitating pathogen persistence.

Within the symptomatic network, *Ralstonia* formed 19 edges, including positive correlations with *Agrobacterium*, *Variovorax*, *Stenotrophomonas*, *Pseudomonas*, *Sphingobium*, and *Nocardioides* (Supplementary Table S2). To capture *Ralstonia* interactions in the asymptomatic group—where it ranked outside the top 50 genera—co-occurrence network analysis was performed on the top 200 genera. This expanded analysis revealed that *Ralstonia* had 54 edges, 25 of which represented negative correlations (Supplementary Table S3). Notably, in the asymptomatic network, *Ralstonia* exhibited negative correlations with *Sphingobium* and *Nocardioides*—genera with which it was positively correlated in the symptomatic network—indicating disease-associated restructuring of these microbial relationships. Several genera exhibiting strong negative correlations with *Ralstonia* are recognized for plant-beneficial or soil-health-promoting functions. These include *Sphingobium* and *Streptomyces*, which are known for aromatic compounds degradation and antimicrobial producing, respectively, as well as *Nocardioides* and *Nitrospira*, which contribute to organic matter decomposition and nitrification.

## 4 Discussion

This study revealed a fundamental shift in understanding tobacco bacterial wilt pathogenesis: rather than merely depleting microbial diversity, the disease drove a targeted restructuring of the rhizosphere microbiome. Metagenomic evidence indicated selective alterations in taxonomic lineages, metabolic pathways, and community interaction networks, converging toward an ecosystem state that favored pathogen persistence.

### 4.1 A selective structural reorganization, not a generalized collapse

The comparable sequencing depth and assembly metrics across all four groups (Tables 1, 2) confirmed that observed differences were biologically driven. This dysbiosis did not manifest as a uniform loss of diversity. Alpha diversity analysis revealed a nuanced pattern of community change. Specifically, observed species richness (Sobs) was consistently lower in asymptomatic than in symptomatic samples at both locations. Notably, the BA group exhibited the lowest mean richness (17,644 ± 582), differing significantly from all other groups. Conversely, community evenness (Shannon and Simpson indices) and overall diversity structure remained statistically unchanged across health states (Table 3).

This pattern suggested that disease acted as a strong selective pressure, reshaping communities through deterministic processes rather than causing stochastic collapse (Berendsen et al., 2012; Mendes et al., 2011). However, this result challenged the prevailing paradigm that pathogen invasion reduces microbial diversity (Qu et al., 2020; Trivedi et al., 2020). The increased richness in symptomatic soils likely reflected disruption of competitive hierarchies, enabling opportunistic taxa to proliferate (Jousset et al., 2017). In asymptomatic soils, competitive exclusion suppresses opportunists; disease disrupts this stability. Notably, the stability of evenness indices indicated that dominant taxa maintained balanced relative abundances despite species turnover. This reflected compensatory proliferation of opportunistic or generalist bacteria (e.g., *Ralstonia*, *Stenotrophomonas*), a phenomenon also documented in *Fusarium*-infected soils (Chapelle et al., 2016). These taxa filled vacant niches, maintaining evenness within simplified communities. Collectively, these findings suggested that dysbiosis involved selective loss of rare taxa and core community reshuffling—a pattern consistent with disturbance theory (Shade et al., 2012).

The pronounced beta-diversity shift, significantly associated with health status, alongside notably higher median Bray-Curtis dissimilarity within the BS group (Figure 2B), underscored that symptomatic communities were not only distinct but also more heterogeneous. This increased within-group variability may reflect two non-mutually exclusive mechanisms. First, it could indicate elevated stochasticity during late-stage disease progression: pathogen invasion can disrupt deterministic assembly processes normally mediated by the host and beneficial microbiota, allowing stochastic events (e.g., random colonization patterns, priority effects) to play a larger role in shaping community composition. This interpretation aligns with the “Anna Karenina principle” proposed for dysbiotic microbiomes, which posits that disturbed communities exhibit greater variability than healthy counterparts (Arnault et al., 2023). Second, the heterogeneity may reflect variation in infection severity or progression stage among individual plants classified as symptomatic based on visual wilt symptoms, which likely represent a continuum from early vascular colonization to systemic infection. Such variation could result in divergent microbial responses, with some communities retaining elements of the native microbiota while others exhibit near-complete turnover (Thomazella et al., 2025). The more pronounced heterogeneity in BS compared to FS might also reflect site-specific differences in agricultural management practices or baseline soil properties, although direct measurements of soil physicochemical parameters were not available in this study to test this hypothesis. We acknowledge that without quantitative pathogen load data, the relative contributions of these mechanisms cannot be definitively resolved. Elucidating these mechanisms will requires longitudinal sampling, as documented in other pathosystems where disease disrupts consistent microbiome assembly (Wei et al., 2019).

### 4.2 Pathogen enrichment and the rise of opportunistic cohorts

Building upon observed structural dysbiosis, our taxonomic profiling at the genus level provided granular insight into specific microbial players. The most unequivocal change was marked and consistent enrichment of the pathogen *R. solanacearum* in symptomatic soils (FS, BS) across both locations, directly confirming its role as the primary disease driver. Beyond the pathogen, a key finding was concomitant enrichment of specific bacterial genera with known pathogenic or opportunistic traits in diseased rhizospheres. Specifically, *Stenotrophomonas* was significantly enriched in symptomatic samples at both locations. Members of this genus are frequently reported as opportunistic pathogens in immunocompromised hosts and often exhibit multidrug resistance (Brooke, 2012), potentially contributing to a more resilient pathogenic consortium. Similarly, *Achromobacter* and *Pseudomonas* exhibited significant enrichment in symptomatic soils at Fenggang and Bozhou, respectively. Although some *Pseudomonas* strains are beneficial, certain species function as opportunistic pathogens in plants, producing virulence factors and thriving in compromised environments (Gao et al., 2014; Vanneste et al., 2011). The co-enrichment of these taxa with *R. solanacearum* aligned with observations from tomato rhizosphere studies, wherein pathogen invasion creates ecological niches favoring fast-growing, generalist bacteria (Wei et al., 2015; Mallon et al., 2015). This suggested that the disease state facilitated proliferation of synergistic or opportunistic cohorts that exploited weakened plant state or benefited from altered rhizosphere chemistry.

Conversely, symptomatic soils exhibited significant and consistent depletion of several putatively beneficial taxa. Genera such as Candidatus_Solibacter, *Luteitalea*, *Pyrinomonas*, and *Methyloceanibacter* were strongly associated with asymptomatic soils. Candidatus_Solibacter and related Acidobacteria are frequently associated with oligotrophic conditions and play important roles in organic matter decomposition and soil carbon cycling (Fierer et al., 2012; Huang et al., 2021; Kielak et al., 2016). Their reduction suggested deterioration of these fundamental soil processes in diseased plots, a phenomenon also documented during *R. solanacearum* invasion of tomato, wherein abundance of multiple bacterial phyla with putative beneficial functions declined sharply (Wei et al., 2018). Furthermore, the decline of genera such as *Metarhizium* (a fungal biomarker for asymptomatic soil) was noteworthy, as this genus comprises species that function as potent insect pathogens and may contribute to indirect plant defense (Hu and Leger, 2002). The loss of such beneficial organisms likely eroded natural disease-suppressive capacity and ecological resilience of the rhizosphere. This pattern mirrored findings that diverse, complex microbial networks, often maintained through a variety of beneficial interactions, are associated with disease suppression, and their simplification constitutes a hallmark of pathogen invasion (Wei et al., 2015; Mendes et al., 2011).

LEfSe biomarker analysis confirmed this contrasting pattern, identifying a conserved set of indicators. *Ralstonia*, *Stenotrophomonas*, and *Pseudoxanthomonas* emerged as common biomarkers of symptomatic soils, forming a potential disease-linked bacterial signature. Conversely, *Candidatus_Solibacter* and *Luteitalea* were robust biomarkers of asymptomatic soils, representing a health-associated microbial profile. This reproducible, site-independent shift highlighted that tobacco bacterial wilt drove a predictable microecological succession characterized by expansion of pathogenic and opportunistic taxa and contraction of key beneficial and stability-associated microorganisms. This trajectory aligned with the theory of alternative stable states, wherein microbiomes exist in either mutualistic (health-associated) or parasitic (disease-associated) configurations, with pathogen invasion serving as a catastrophic shift that restructures the entire ecosystem (Fukami, 2015; Fukami and Nakajima, 2011). The consistency of this shift across two distinct geographical locations suggested that tobacco bacterial wilt imposed strong deterministic selection, overriding local edaphic variability to converge on a functionally degraded, pathogen-permissive state. This aligned with the broader ecological concept that pathogen invasions can trigger predictable and detrimental shifts in host-associated microbiomes, reducing functional potential (Wei et al., 2018; Chapelle et al., 2016).

### 4.3 Functional reprograming toward a stressed, competitive state

Functional metagenomic analysis revealed that tobacco bacterial wilt drove a metabolic shift from balanced growth toward stress survival and competition, characterized by widespread increases in KEGG functional gene representation in symptomatic soils. This increase across most KEGG categories indicated heightened metabolic potential and genetic redundancy. This pattern was attributed to two main factors. First, proliferation of diverse opportunistic bacteria (e.g., *Stenotrophomonas*, *Pseudomonas*), as identified in our taxonomic analysis, directly introduced a broad suite of functional genes into the community pool (Silby et al., 2011). Second, and more critically, the diseased rhizosphere represented a highly stressful and resource-competitive environment. The exclusive enrichment of functional modules related to cell motility, transport and catabolism, and cellular community in symptomatic samples reflected a microbial community intensely engaged in niche exploration, substrate scavenging, and intercellular interactions—hallmarks of communities under severe abiotic and biotic stress (Shade et al., 2012).

COG classification further revealed strategic reprioritization of metabolic investment under disease pressure. We observed a marked increase in genes associated with defense mechanisms in the symptomatic microbiome. This pronounced upregulation indicated community-wide mobilization of antagonistic and stress-resistance traits, consistent with the dramatic increase in antibiotic resistance genes (ARGs) identified by CARD analysis. The observed ARG enrichment may stem from two contributing sources. First, these ARGs may be primarily carried by *Ralstonia solanacearum*, which harbors multiple ARGs (Li et al., 2024). Given the significant enrichment of *Ralstonia* in symptomatic soils (Figure 4), pathogen proliferation likely contributed to increased ARG load. Second, elevated ARGs may reflect a broader community-level stress response to pathogen invasion. Root exudates and rhizosphere metabolites regulate ARG dynamics in plant-associated microbiomes (Tan et al., 2025; Han et al., 2025), with plant growth-promoting bacteria potentially serving as ARG reservoirs (Youseif et al., 2025). The community-level stress response mechanism was further supported by COG functional analysis. Notably, while core housekeeping functions such as amino acid transport and metabolism also increased, their relative enrichment was less pronounced. This pattern suggested that in the dysbiotic state, the microbial community strategically diverted genomic investment toward survival and antagonism, potentially at the expense of optimizing core anabolic processes for cooperative stability (Wei et al., 2015). This did not reflect simple shutdown of primary metabolism but rather a calculated shift in functional emphasis toward traits conferring immediate fitness in a hostile, competitive niche. We acknowledge that without host assignment through binning or characterization of mobile genetic elements, the relative contributions of these mechanisms cannot be definitively resolved. Future studies employing metagenomic binning could identify ARG hosts and assess horizontal transfer potential. Nevertheless, our findings contribute to the emerging understanding that soil-borne disease outbreaks are accompanied by increased antibiotic resistance in agricultural ecosystems (Li et al., 2024).

This core metabolic shift was compounded by other functional signatures. The significant enrichment of genes encoding Carbohydrate-Active Enzymes (CAZy), particularly glycoside hydrolases (GHs), indicated a community with enhanced potential for aggressive nutrient scavenging from decaying plant tissues, a common response to pathogen-induced root damage that intensified competition for carbon (Chapelle et al., 2016). Collectively, these functional changes revealed a consistent pattern: the diseased rhizosphere harbored a microbiome whose genetic repertoire was reconfigured for combative, exploitative ecological strategies. This collective reprograming away from mutualistic or stable metabolic networks and toward a competitive survival functional state likely undermined the very ecosystem services—such as nutrient cycling stability—that characterized a healthy rhizosphere. This may have created a self-reinforcing feedback loop, wherein heightened competition further eroded community cooperation and structure, ultimately cementing the dysbiotic state and facilitating pathogen persistence (Jousset et al., 2017).

### 4.4 Network destabilization: erosion of competitive constraints

Co-occurrence network analysis provided a topological perspective revealing the ecological consequences of observed dysbiosis. The asymptomatic rhizosphere harbored a microbial network with greater overall connectivity and a notably higher proportion of negative correlations. This architecture reflected a stable, mature community wherein dense antagonistic interactions created a web of mutual constraints, a structure theorized to suppress pathogen proliferation through “growth suppression” and characteristic of disease-suppressive soils (Wei et al., 2015). Conversely, the symptomatic network exhibited reduced connectivity and fewer negative interactions, indicating relaxation of competitive pressures and loss of ecological resilience (Shade et al., 2012). This erosion of competitive constraints was further evidenced by profound rewiring of *R. solanacearum* interactions: from negatively correlations with beneficial taxa such as *Sphingobium* in healthy soils to positive correlations in diseased soils, representing a critical breakdown of microbial antagonism.

Concurrently, the network core underwent functional restructuring. The asymptomatic network was anchored by beneficial hubs characterized by antagonism and plant growth promotion (e.g., *Pseudomonas*, *Arthrobacter*), forming a coherent suppressive consortium. This structure was replaced in the symptomatic state by new, highly connected genera such as *Streptomyces*. Their rise in simplified network reflected stressed community forming alternative, potentially less effective, defensive alliances, consistent with the functional shift toward a stressed, combative state. This reconfiguration of keystone taxa, together with pathogen integration into a web of positive associations, signified fundamental restructuring of the community’s ecological core.

In summary, bacterial wilt drove a transition from a complex, competition-stabilized state to a simplified, competition-released state. The erosion of competitive constraints and pathogen integration into the reorganized community created a permissive ecological niche that facilitated its persistence. This topological collapse represented the ultimate manifestation of rhizosphere microbiome dysbiosis, wherein the loss of structural integrity undermined the community’s capacity to resist invasion and maintain ecosystem health.

## 5 Conclusion

This study demonstrated that tobacco bacterial wilt induced a systemic dysbiosis of the rhizosphere microbiome, characterized by three core changes. First, disease caused a selective taxonomic reshuffling, significantly increasing species richness in symptomatic soils and consistently enriching the pathogen *Ralstonia* and opportunistic genera such as *Stenotrophomonas*, while depleting beneficial taxa including Candidatus_Solibacter, *Luteitalea*, and *Metarhizium*. Second, this shift drove collective metabolic reprogramming toward a stressed, competitive state, as evidenced by a marked increase in genes for carbohydrate scavenging (e.g., Glycoside Hydrolases: 14,466 vs. 13,847) and dramatic proliferation of antibiotic resistance gene categories (27 vs. 2). Third, co-occurrence network analysis revealed ecological collapse underlying this shift: the healthy microbiome’s complex and competitive network (665 edges, high proportion of negative correlations) was simplified and destabilized in symptomatic soil (604 edges), with pathogen interactions rewired from negative to positive correlations with key taxa. Collectively, these multi-level disruptions confirmed that disease progression represented an ecological cascade that eroded microbiome stability and suppressive capacity, identifying specific microbial indicators and network properties as targets for disease diagnosis and microbiome-based management.

## Data Availability

The data presented in the study are deposited in the NCBI repository, accession number PRJNA1403154.

## Footnotes

1
https://www.illumina.com

2
https://www.majorbio.com

3
https://github.com/OpenGene/fastp

4
https://github.com/voutcn/megahit

5
https://github.com/hyattpd/Prodigal

6
https://weizhongli-lab.org/cd-hit/

7
https://github.com/ShujiaHuang/SOAPaligner

8
https://ab.inf.uni-tuebingen.de/software/diamond/

9
https://cran.r-project.org/web/packages/vegan/index.html
