# Supplementary material for: Metagenomic insights into the rhizosphere microbiome dysbiosis associated with tobacco bacterial wilt
Source: Front Microbiol. 2026 Apr 15;17:1809980. doi: 10.3389/fmicb.2026.1809980 (PMC13124702; doi:10.3389/fmicb.2026.1809980)
Supplement: Supplementary file 1 [file Table_1.docx]

Supplementary Table S1. Network topological properties of the top 50 abundant bacterial genera in symptomatic and asymptomatic rhizosphere soils

| Node_Name | Symptomatic group | | Asymptomatic group | |
| --- | --- | --- | --- | --- |
|  | Degree | Clustering | Degree | Clustering |
| unclassified_p__Candidatus_  Methylomirabilota | 34 | 0.79679 | 35 | 0.89916 |
| *Nitrospira* | 32 | 0.79839 | 17 | 0.67647 |
| *Gemmatimonas* | 30 | 0.83908 | 34 | 0.91622 |
| *Nocardioides* | 35 | 0.74454 | 37 | 0.78378 |
| *Ralstonia* | 19 | 0.61988 | — | — |
| Candidatus_Defluviilinea | 27 | 0.82621 | 5 | 1 |
| *Gemmatirosa* | 32 | 0.80444 | 33 | 0.91856 |
| *Gaiella* | 34 | 0.76471 | 34 | 0.90731 |
| *Sphingomonas* | 29 | 0.80296 | 31 | 0.94839 |
| *Chloracidobacterium* | 23 | 0.79842 | 33 | 0.71023 |
| Candidatus_Villigracilis | 33 | 0.82197 | 12 | 0.81818 |
| unclassified_p__Candidatus_  Dormiibacterota | 31 | 0.77419 | 36 | 0.83651 |
| *Pyrinomonas* | 30 | 0.8023 | 35 | 0.71429 |
| unclassified_f__Nitrososphaeraceae | 36 | 0.7381 | 24 | 0.88768 |
| unclassified_p__Candidatus_  Rokuibacteriota | 34 | 0.79857 | 24 | 1 |
| *Longimicrobium* | 32 | 0.80444 | 34 | 0.91622 |
| *Roseisolibacter* | 30 | 0.83908 | 35 | 0.89916 |
| unclassified_o__Candidatus_  Saccharimonadales | 29 | 0.78325 | 32 | 0.83669 |
| *Pedococcus* | 31 | 0.76344 | 32 | 0.87702 |
| *Usitatibacter* | 31 | 0.8129 | 35 | 0.78992 |
| *Anaerolinea* | 30 | 0.82989 | 35 | 0.82857 |
| *Pseudomonas* | 19 | 0.95322 | — | — |
| Candidatus_Udaeobacter | 28 | 0.78836 | 30 | 0.92414 |
| *Sphingobium* | 15 | 0.95238 | 36 | 0.75397 |
| *Chryseobacterium* | 33 | 0.8125 | — | — |
| *Trebonia* | 26 | 0.86462 | 33 | 0.74432 |
| *Ilumatobacter* | 32 | 0.75605 | 33 | 0.71023 |
| *Arthrobacter* | 36 | 0.7381 | — | — |
| *Anaeromyxobacter* | 33 | 0.81439 | 35 | 0.87227 |
| *Candidatus_Solibacter* | 34 | 0.76649 | 35 | 0.89916 |
| *Mesorhizobium* | 32 | 0.75806 | — | — |
| *Chitinophaga* | 31 | 0.80645 | — | — |
| *Variovorax* | 36 | 0.77619 | — | — |
| *Agrobacterium* | 28 | 0.78571 | — | — |
| *Ktedonobacter* | 29 | 0.75369 | — | — |
| unclassified_c__Candidatus_Binatia | 28 | 0.70635 | 26 | 0.59692 |
| *Luteitalea* | 15 | 0.73333 | 32 | 0.875 |
| *Achromobacter* | 18 | 0.50327 | — | — |
| *Methyloceanibacter* | 13 | 0.70513 | 36 | 0.83333 |
| *Kouleothrix* | 5 | 0.9 | 34 | 0.89305 |
| *Sphingomicrobium* | 23 | 0.82609 | 36 | 0.84921 |
| *Streptomyces* | 13 | 0.73077 | 36 | 0.82063 |
| *Bradyrhizobium* | 2 | 1 | 5 | 0.6 |
| *Reyranella* | 9 | 0.80556 | 4 | 0.83333 |
| *Stenotrophomonas* | 5 | 0.9 | — | — |
| Candidatus_Acidiferrum | 6 | 0.73333 | 8 | 0.67857 |
| unclassified_p__Candidatus_  Eiseniibacteriota | 6 | 0.73333 | 2 | 1 |
| Candidatus_Sulfotelmatobacter | 5 | 0.9 | 33 | 0.88068 |
| Candidatus_Angelobacter | 5 | 0.8 | 9 | 0.75 |
| *Pseudoxanthomonas* | 1 | 0 | — | — |
| *Sphingomicrobium* | — | — | 36 | 0.84921 |
| *Conexibacter* | — | — | 34 | 0.90731 |
| *Pseudolabrys* | — | — | 2 | 0 |
| *Micromonospora* | — | — | 37 | 0.83033 |
| Candidatus_Ribeiella | — | — | 37 | 0.84835 |
| Candidatus_Sulfopaludibacter | — | — | 33 | 0.91856 |
| *Mycobacterium* | — | — | 4 | 0.83333 |
| *Flavisolibacter* | — | — | 9 | 0.69444 |
| Candidatus_Deferrimicrobium | — | — | 30 | 0.7977 |
| Candidatus_Elarobacter | — | — | 34 | 0.90374 |
| *Aromatoleum* | — | — | 4 | 1 |
| unclassified_c__Candidatus_  Bathyarchaeia | — | — | 37 | 0.84835 |
| *Intrasporangium* | — | — | 13 | 0.53846 |

Note: Network topological parameters were calculated for the top 50 most abundant bacterial genera based on co-occurrence networks constructed using Spearman's rank correlations (|r| > 0.7, *p* < 0.05). "—" indicates absence from the network (degree = 0).

Supplementary Table S2. Spearman's rank correlations between *Ralstonia* and co-occurring bacterial genera in symptomatic rhizosphere soils

| Node1_Name | Node2_Name | Coefficient | Pvalue |
| --- | --- | --- | --- |
| *Ralstonia* | *Agrobacterium* | 0.76969697 | 0.009221953 |
| *Ralstonia* | *Variovorax* | 0.784848485 | 0.028882798 |
| *Ralstonia* | *Stenotrophomonas* | 0.784848485 | 0.028882798 |
| *Ralstonia* | *Pseudomonas* | 0.866666667 | 0.001173538 |
| *Ralstonia* | *Sphingobium* | 0.757575758 | 0.011143447 |
| *Ralstonia* | *Nocardioides* | 0.736363636 | 0.047911726 |
| *Ralstonia* | *Ilumatobacter* | -0.736363636 | 0.047911726 |
| *Ralstonia* | Candidatus_Defluviilinea | -0.760606061 | 0.037588378 |
| *Ralstonia* | unclassified_p__Candidatus_  Dormiibacterota | -0.721212121 | 0.018573155 |
| *Ralstonia* | Candidatus_Acidiferrum | -0.806060606 | 0.004862061 |
| *Ralstonia* | unclassified_p__Candidatus_  Eiseniibacteriota | -0.748484848 | 0.042540128 |
| *Ralstonia* | Candidatus_Sulfotelmatobacter | -0.784848485 | 0.028882798 |
| *Ralstonia* | unclassified_o__Candidatus_  Saccharimonadales | -0.793939394 | 0.006099923 |
| *Ralstonia* | *Usitatibacter* | -0.772727273 | 0.033041223 |
| *Ralstonia* | Candidatus_Udaeobacter | -0.757575758 | 0.011143447 |
| *Ralstonia* | unclassified_p__Candidatus_  Rokuibacteriota | -0.781818182 | 0.007547008 |
| *Ralstonia* | *Anaeromyxobacter* | -0.745454545 | 0.013330146 |
| *Ralstonia* | Candidatus_Solibacter | -0.793939394 | 0.006099923 |
| *Ralstonia* | unclassified_p__Candidatus_  Methylomirabilota | -0.733333333 | 0.015800596 |

Supplementary Table S3. Spearman's rank correlations between *Ralstonia* and bacterial genera in asymptomatic rhizosphere soils (expanded network analysis)

| Node1_Name | Node2_Name | Coefficient | Pvalue |
| --- | --- | --- | --- |
| *Ralstonia* | *Ktedonobacter* | 0.781818182 | 0.007547008 |
| *Ralstonia* | *Nitrolancea* | 0.781818182 | 0.007547008 |
| *Ralstonia* | Candidatus_Leptofilum | 0.721212121 | 0.018573155 |
| *Ralstonia* | *Paenibacillus* | 0.709090909 | 0.021665923 |
| *Ralstonia* | *Noviherbaspirillum* | 0.772727273 | 0.033041223 |
| *Ralstonia* | Candidatus_Deferrimicrobium | 0.748484848 | 0.042540128 |
| *Ralstonia* | *Phenylobacterium* | 0.866666667 | 0.001173538 |
| *Ralstonia* | *Caldilinea* | 0.793939394 | 0.006099923 |
| *Ralstonia* | unclassified_p__Candidatus_  Zixiibacteriota | 0.748484848 | 0.042540128 |
| *Ralstonia* | *Longilinea* | 0.772727273 | 0.033041223 |
| *Ralstonia* | *Thermoflexus* | 0.748484848 | 0.042540128 |
| *Ralstonia* | *Labilithrix* | 0.748484848 | 0.042540128 |
| *Ralstonia* | *Bellilinea* | 0.709090909 | 0.021665923 |
| *Ralstonia* | *Sorangium* | 0.757575758 | 0.011143447 |
| *Ralstonia* | Candidatus_Promineifilum | 0.781818182 | 0.007547008 |
| *Ralstonia* | *Ktedonosporobacter* | 0.806060606 | 0.004862061 |
| *Ralstonia* | Candidatus_Desulfolinea | 0.757575758 | 0.011143447 |
| *Ralstonia* | *Polyangium* | 0.709090909 | 0.021665923 |
| *Ralstonia* | *Ardenticatena* | 0.733333333 | 0.015800596 |
| *Ralstonia* | *Litorilinea* | 0.757575758 | 0.011143447 |
| *Ralstonia* | *Caulobacter* | 0.793939394 | 0.006099923 |
| *Ralstonia* | *Dictyobacter* | 0.866666667 | 0.001173538 |
| *Ralstonia* | Candidatus_Roseilinea | 0.781818182 | 0.007547008 |
| *Ralstonia* | Candidatus_Leptovillus | 0.745454545 | 0.013330146 |
| *Ralstonia* | Candidatus_Amarolinea | 0.781818182 | 0.007547008 |
| *Ralstonia* | *Thermanaerothrix* | 0.745454545 | 0.013330146 |
| *Ralstonia* | *Levilinea* | 0.796969697 | 0.025096676 |
| *Ralstonia* | *Sphaerobacter* | 0.83030303 | 0.002940227 |
| *Ralstonia* | Candidatus_Villigracilis | 0.733333333 | 0.015800596 |
| *Ralstonia* | unclassified_p__Candidatus_  Neomarinimicrobiota | -0.793939394 | 0.006099923 |
| *Ralstonia* | *Escherichia* | -0.772727273 | 0.033041223 |
| *Ralstonia* | *Luteitalea* | -0.83030303 | 0.002940227 |
| *Ralstonia* | *Nocardioides* | -0.784848485 | 0.028882798 |
| *Ralstonia* | *Pyrinomonas* | -0.733333333 | 0.015800596 |
| *Ralstonia* | unclassified_f__  Nitrososphaeraceae | -0.83030303 | 0.002940227 |
| *Ralstonia* | *Chloracidobacterium* | -0.772727273 | 0.033041223 |
| *Ralstonia* | *Sphingobium* | -0.842424242 | 0.002220031 |
| *Ralstonia* | *Streptomyces* | -0.772727273 | 0.033041223 |
| *Ralstonia* | *Rhizorhapis* | -0.781818182 | 0.007547008 |
| *Ralstonia* | *Marmoricola* | -0.796969697 | 0.025096676 |
| *Ralstonia* | *Steroidobacter* | -0.796969697 | 0.025096676 |
| *Ralstonia* | *Pseudolysinimonas* | -0.733333333 | 0.015800596 |
| *Ralstonia* | unclassified_p__Thermoproteota | -0.806060606 | 0.004862061 |
| *Ralstonia* | *Povalibacter* | -0.796969697 | 0.025096676 |
| *Ralstonia* | unclassified_c__Candidatus_  Saccharimonadia | -0.83030303 | 0.002940227 |
| *Ralstonia* | *Novosphingobium* | -0.736363636 | 0.047911726 |
| *Ralstonia* | *Acidobacterium* | -0.818181818 | 0.00381492 |
| *Ralstonia* | *Aridibacter* | -0.721212121 | 0.018573155 |
| *Ralstonia* | *Humisphaera* | -0.748484848 | 0.042540128 |
| *Ralstonia* | Candidatus_Cybelea | -0.842424242 | 0.002220031 |
| *Ralstonia* | *Microlunatus* | -0.748484848 | 0.042540128 |
| *Ralstonia* | unclassified_c__Candidatus_  Binatia | -0.709090909 | 0.021665923 |
| *Ralstonia* | *Nitrospira* | -0.709090909 | 0.021665923 |
| *Ralstonia* | unclassified_p__Candidatus_  Tectimicrobiota | -0.772727273 | 0.033041223 |
